# Supplementary material for: A fault-intrusion-tolerant system and deadline-aware algorithm for scheduling scientific workflow in the cloud
Source: PeerJ Comput Sci. 2021 Nov 2;7:e747. doi: 10.7717/peerj-cs.747 (PMC8576554; doi:10.7717/peerj-cs.747)
Supplement: Supplemental Information 6 [file peerj-cs-07-747-s006.zip › Myproject1.1/docs/org/cloudbus/cloudsim/examples/power/random/package-frame.html]

org.cloudbus.cloudsim.examples.power.random


# org.cloudbus.cloudsim.examples.power.random

## Classes

- Dvfs
- IqrMc
- IqrMmt
- IqrMu
- IqrRs
- LrMc
- LrMmt
- LrMu
- LrrMc
- LrrMmt
- LrrMu
- LrrRs
- LrRs
- MadMc
- MadMmt
- MadMu
- MadRs
- NonPowerAware
- RandomConstants
- RandomHelper
- RandomRunner
- ThrMc
- ThrMmt
- ThrMu
- ThrRs
